# Multimodal imaging for early drusen detection: RM-SLO as a promising imaging approach

**DOI:** 10.3389/fmed.2025.1703332

**Published:** 2026-01-12

**Authors:** Shujun Zhang, Xiaoling Zhang, Zixun Wang, Wei Shi, Zhaoyang Ren, Yanna Wang, Qian Jia, Yongchao Li, Yali Guo, Zhiqing Li

**Affiliations:** 1 Handan Eye Hospital (The Third Hospital of Handan), Hebei, China; 2 Tianjin Key Laboratory of Retinal Functions and Diseases, Tianjin Branch of National Clinical Research Center for Ocular Disease, Eye Institute and School of Optometry, Tianjin Medical University Eye Hospital, Tianjin, China

**Keywords:** age-related macular degeneration, drusen, early detection, multimodal imaging, retro-mode scanning laser ophthalmoscopy

## Abstract

**Purpose:**

This study aimed to evaluate the diagnostic performance of retro-mode scanning laser ophthalmoscopy (RM-SLO) in detecting drusen compared to conventional multimodal imaging. Additionally, it sought to explore the imaging characteristics of RM-SLO for early age-related macular degeneration (AMD).

**Methods:**

In this cross-sectional study, 192 patients (263 eyes) were examined using color fundus photography (CFP), optical coherence tomography (OCT), fundus fluorescein angiography (FFA), fundus autofluorescence (FAF), and RM-SLO. Three retinal specialists independently reviewed all images for the presence of drusen. Detection rates across modalities were compared using Cochran’s Q-test, and imaging characteristics of hard and soft drusen were described.

**Results:**

RM-SLO detected drusen in 102 eyes (38.78%). This detection rate was significantly higher compared to other methods such as OCT (22.05%), CFP (8.37%), FFA (5.32%), and FAF (4.18%) (all *p* < 0.0001 vs. RM-SLO). OCT was superior to CFP, FAF, and FFA (*p* < 0.0001); CFP had statistically significant differences from FFA and FAF (*p* = 0.039; *p* = 0.001). While FFA did not reveal any statistically significant differences from FAF (*p* = 0.453). RM-SLO provided a clear pseudo-three-dimensional visualization, enabling the identification of both hard and soft drusen, including small lesions that were not captured by other modalities.

**Conclusion:**

RM-SLO demonstrates superior sensitivity and imaging clarity for drusen detection compared to conventional multimodal approaches. Its ability to visualize small and morphologically distinct drusen highlights its potential as a promising tool for early AMD screening and clinical management.

## Introduction

Age-related macular degeneration (AMD) can be one of the most common causes of visual impairment and blindness in older patients, increasing the burden on social care and support services (1–3). It has become increasingly important as a public health issue due to the aging population and greater life expectancy (4, 5). Through early identification and diagnosis, early intervention treatment can be effective (6–8). Identifying early imaging markers for AMD is a critical step in facilitating timely detection and intervention (9).

Drusen is one of the most characteristic pathologic manifestations of AMD precursor lesions and is an important factor in the development of AMD (10, 11). The drusen consist of extracellular deposits of lipids, proteins, and cellular debris present between the basal layer of the retinal pigment epithelium (RPE) and the inner layer of Bruch’s membrane (BM) (12), which appear as small yellow deposits on fundoscopy (13). Current methods of diagnosing drusen include color fundus photography(CFP), optical coherence tomography (OCT), and fluorescein fundus angiography (FFA) (14). Fundus autofluorescence (FAF) is a type of non-invasive fundus examination that can show the content and distribution of lipofuscin in the RPE and reflects the function and metabolism of RPE cells (15). Scanning laser ophthalmoscopy in the retro-mode (RM-SLO) fundus imaging is an imaging method used in recent years (16). By restricting the opening of the annular aperture so that it deviates laterally from the confocal path from the left or right side, respectively, only scattered light from one direction is collected, and scattered light from other directions is blocked to form shadows, and under the scanning of a high-penetrating infrared laser, RPE, neural epithelium, and deep retinal structures are imaged by high-contrast imaging. Under high-penetrating IR laser scanning, the RPE, neuroepithelium, and deeper retinal structures are imaged with high contrast, resulting in a clear pseudo three-dimensional image (17, 18).

Regardless of the imaging modality, several studies have investigated its effectiveness in detecting different fundus lesions. CFP offers convenient and objective features but has limited effectiveness in detecting deep lesions in the early retina (19). Correspondingly, OCT, by longitudinal scanning, has significant advantages in measuring the size and boundaries of drusen and evaluating imaging biomarkers related to drusen structure; however, since OCT can only show the longitudinal structure of the retina, it is not possible to visualize the morphology and area of drusen (20). FFA is commonly used to assess the extent and nature of lesions, and this dynamic imaging method allows the study of retinal perfusion and the binding properties of different retinal layers to fluorescein dye to reflect the nature of the lesion. However, it is invasive and uses sodium fluorescein as the dye, and some patients are at risk of an allergy to the contrast medium (21). Lv et al. (22) utilized RM-SLO to study the detectability of diabetic retinopathy and compared OCT and CFP with RM-SLO (22). Although only a limited number of comparative studies between RM-SLO and other imaging modalities have been reported (22–26), the majority of these focused on diabetic retinopathy or AMD subtypes rather than a direct, systematic comparison for drusen detection. Therefore, comprehensive comparative data in this specific context remain scarce, which motivated our current study.

Therefore, this study aimed to observe the detection capabilities of CFP, OCT, RM-SLO, FFA, and FAF fundus imaging in identifying drusen lesions and to analyze the imaging characteristics of RM-SLO in drusen detection.

## Methods

### Data collection and ethics statement

**Figure 1:**
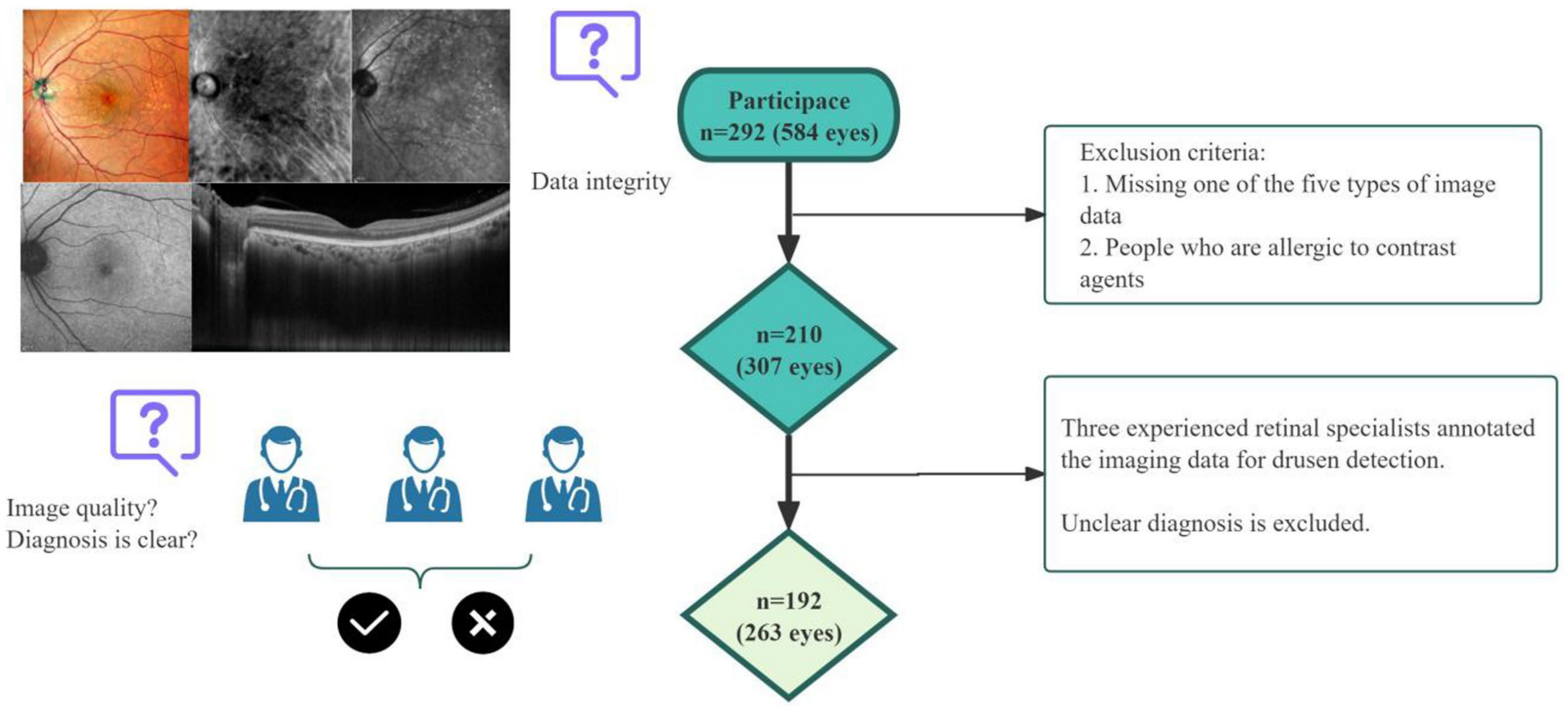
Design process of our study. A total of 292 patients (584 eyes) were recruited for the research. Before enrollment, 82 cases (277 eyes) were excluded due to missing 1 or more image data or refusal to participate. Three experienced retinal specialists annotated the imaging data for drusen detection. A total of 18 patients (44 eyes) with unclear diagnoses were excluded.

This study was approved by the Ethics Committee of Handan Eye Hospital (The Third Hospital of Handan) (No. 2025005) and conducted in accordance with the guiding principles of the Declaration of Helsinki. This cross-sectional study was conducted involving 292 patients with dry eye disease and/or cataract surgery patients who felt ocular discomfort and were treated at Handan Eye Hospital (The Third Hospital of Handan) from July 2024 to August 2025. Patients were excluded from the study if they or their family members did not obtain consent to participate in the study. Patients who had the following conditions were also excluded: (1) presence of refractive media opacity that affected the quality of image acquisition; (2) history of previous retinal or macular surgery, laser treatment, or injections, as well as a history of eye trauma; (3) congenital macular diseases; (4) failure to undergo the required examination; and (5) other fundus diseases. A total of 210 patients (307 eyes) were included. Three experienced retinal experts independently diagnosed the image data for drusen detection. After the independent evaluation was completed, for the cases in which the opinions of the three experts were inconsistent, we adopted the method of determining the diagnosis result by the senior expert, and then the other two experts re-examined the image data and discussed it together. Finally, 18 patients (44 eyes) were excluded due to the diagnosis being still controversial and unclear. Ultimately, the analysis included 263 eyes from 192 patients with consensus evaluations by all 3 experts (Figure 1).

All patients underwent an electrocardiogram, blood pressure measurement, biochemical tests, and other assessments, and there were no contraindications identified for the FFA examination. To ensure the clarity of the image, mydriasis was performed in advance, and the diameter of the bilateral pupils was greater than 4 mm. The confocal laser fundus angiography instrument (HRA-2, Heidelberg Company, Germany) was used, and all patients underwent FAF and FFA examinations with the macula as the center. FAF and FFA used a blue excitation light source with a wavelength of 488 nm and a collection range of 55°, and clear fundus images were collected and saved for analysis. OCT (OCTARS-3000 Advance, Nidek, Japan) selected the Macular Cube 512 × 128 mode to scan an area of 12 mm × 9 mm centered on the macula with a scanning density of 512 × 128 B-scan. Wide-field laser fundus imaging was performed using RM-SLO (Mirante SLO, Nidek, Japan), including fundus imaging of the right retro illumination (RMDR), left retro illumination (RMDL), and annular ring aperture (RMDA), with a wavelength of 790 nm and a collection range of 89°. The images produced with RMDL and RMDA lack clarity, so we selected the image generated by RMDR for the research.

### Statistical analysis

Continuous variables were tested for normality using the Shapiro–Wilk (S–W) test. Data that were normally distributed were presented as mean ± SD, while non-normal distributions were shown as median [P_25_, P_75_]. Categorical variables were presented as counts and percentages, and the results were analyzed using SPSS 21.0 software (IBM, New York, USA). Statistical analysis among groups was performed using the exact Cochran’s *Q*-test, with post-hoc pairwise comparisons conducted using Bonferroni-corrected multiple McNemar’s tests. When the expected frequency was <5, an exact binomial test was used instead. A *p*-value of < 0.05 was considered statistically significant.

## Results

As shown in Table 1, of the 192 patients included, 89 (46.35%) were men, and 103 (53.65%) were women, with an age of 54 [40, 62] years. Among the 263 eyes included, 132 (50.19%) were right eyes and 131 (49.81%) were left eyes. After diagnosed by 3 experienced retinal physicians, 102 eyes (38.78%) were diagnosed for drusen by RM-SLO, 58 eyes (22.05%) by OCT, 14 eyes (5.32%) by FFA, 11 eyes (4.18%) by FAF, and 22 cases (8.37%) by CFP.

**Table 1:** Detection of drusen by RM-SLO, OCT, FFA, FAF, and CFP was analyzed, and the baseline characteristics of the included patients.

Basic information	Total	RM-SLO	OCT	FFA	FAF	CFP
Number of eyes	263	102 (38.78%)	58 (22.05%)	14 (5.32%)	11 (4.18%)	22 (8.37%)
Laterality						
OD, %	132, 50.19%	53, 51.96%	32, 55.17%	5, 35.71%	3, 27.27%	10, 45.45%
OS, %	131, 49.81%	49, 48.04%	26, 44.83%	9, 64.29%	8, 72.73%	12, 54.55%
Number of patients	192	90	49	13	9	18
Gender						
Men, %	89, 46.35%	32, 35.56%	14, 28.57%	4, 30.77%	1, 11.11%	3, 16.67%
Women, %	103, 53.65%	58, 64.44%	35, 71.43%	9, 69.23%	8, 88.89%	15, 83.33%
Age, mean ± SD, y; (*p*-value)	54 [40, 62] (0.000)	60.30 ± 9.90 (0.058)	62 [59, 68] (0.036)	62.31 ± 13.43 (0.443)	62.11 ± 16.34 (0.132)	63.11 ± 11.81 (0.237)

**Figure 2:**
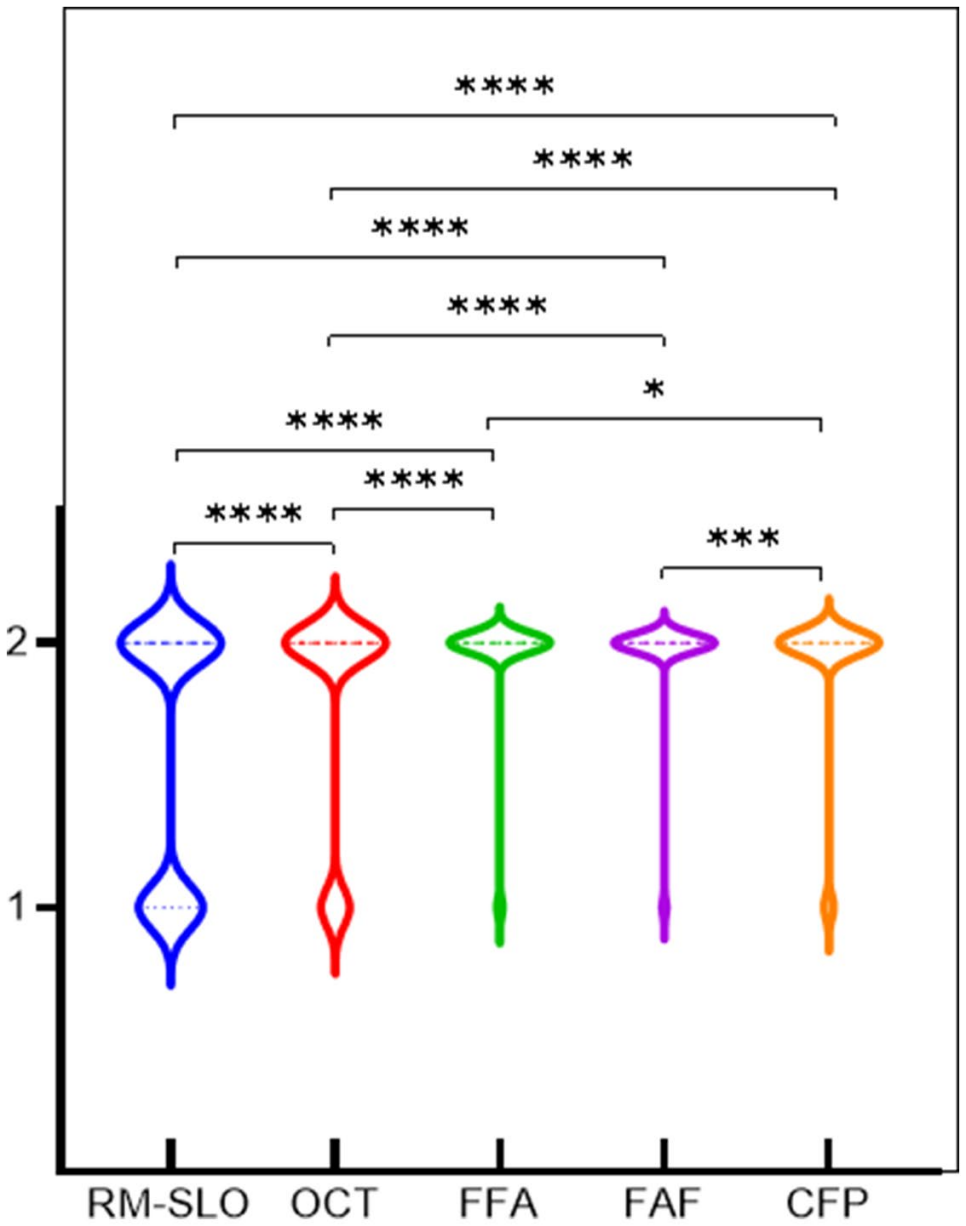
Differences between different detection equipment. 1 and 2 represent the diagnosis of drusen and not being diagnosed as having drusen.

The detection distribution and differences among various detection equipment are illustrated in Figure 2 and Table 2. Particularly, when compared to OCT, FFA, FAF, and CFP, RM-SLO showed statistically significant detection results (*p* < 0.0001). OCT demonstrated statistically significant detection results in comparison to FFA, FAF, and CFP (*p* < 0.0001). CFP had statistically significant differences between FFA and FAF (*p* = 0.039; *p* = 0.001). On the other hand, FFA did not reveal any statistically significant differences from FAF (*p* = 0.453).

**Table 2:** Analysis of the differences between different detection equipment.

Detection equipment	RM-SLO	OCT	FFA	FAF	CFP
RM-SLO	—				
OCT	<0.0001	—			
FFA	<0.0001	<0.0001	—		
FAF	<0.0001	<0.0001	0.453^*^	—	
CFP	<0.0001	<0.0001	0.039^*^	0.001^*^	—

^*^The exact binomial test was applied due to the small sample size; other comparisons used. McNemar’s test with Bonferroni correction.

**Figure 3:**
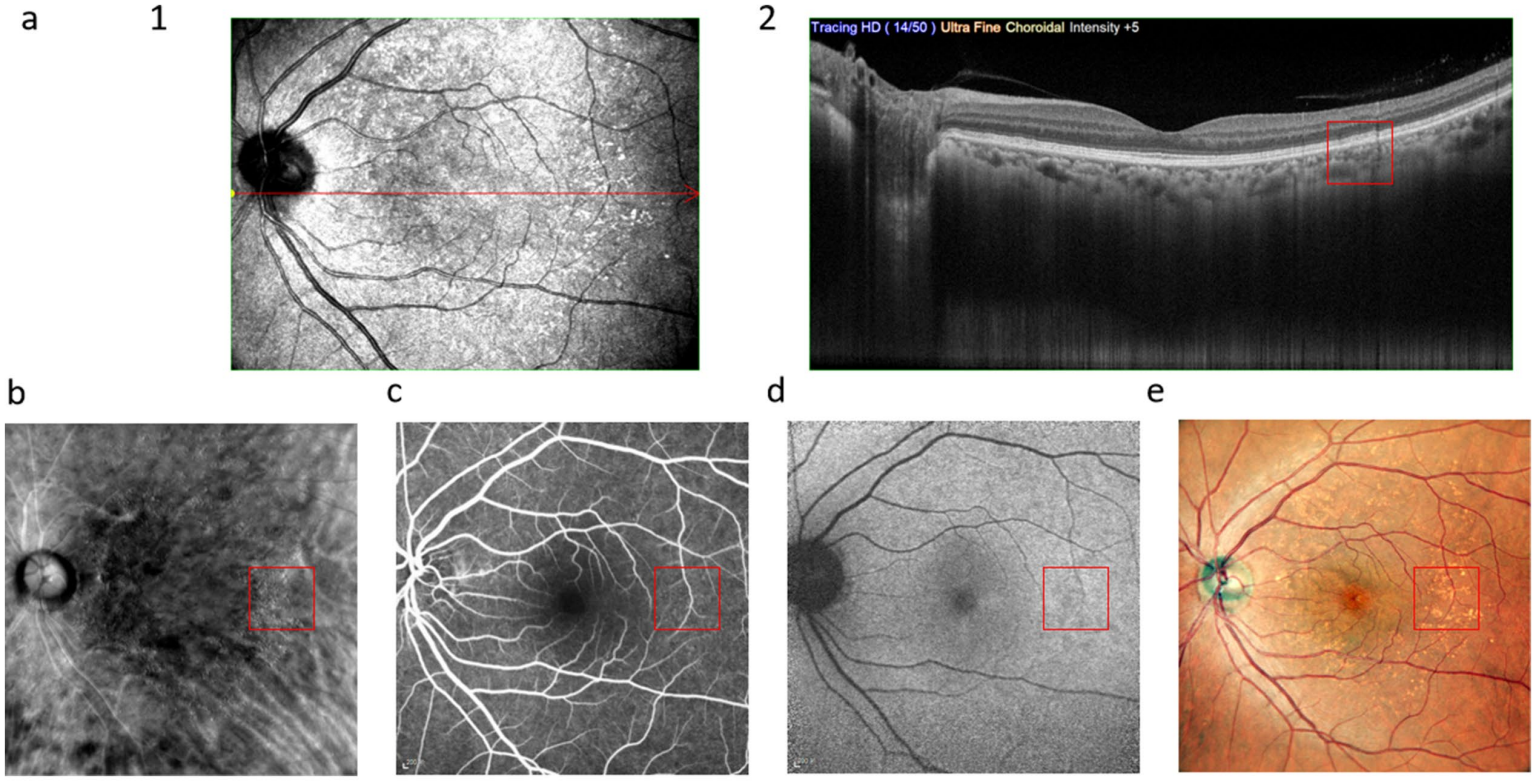
Multimodal images of hard drusen: **(a)**, (1) infrared scan, where the red line is the OCT scanning position of (2); **(b)**, RM-SLO in RMDR directions; **(c)**, FFA; **(d)**, FAF; **(e)**, CFP. The red box shows the drusen.

As shown in Figure 3, the hard drusen present as focal dome-shaped RPE-Bruch membrane complexes on OCT. The RM-SLO along the RMDR direction shows small elevations, while FFA reveals medium-term persistent high fluorescence with clear borders. FAF demonstrates high autofluorescence, and CFP exhibits small, round yellow dot lesions.

**Figure 4:**
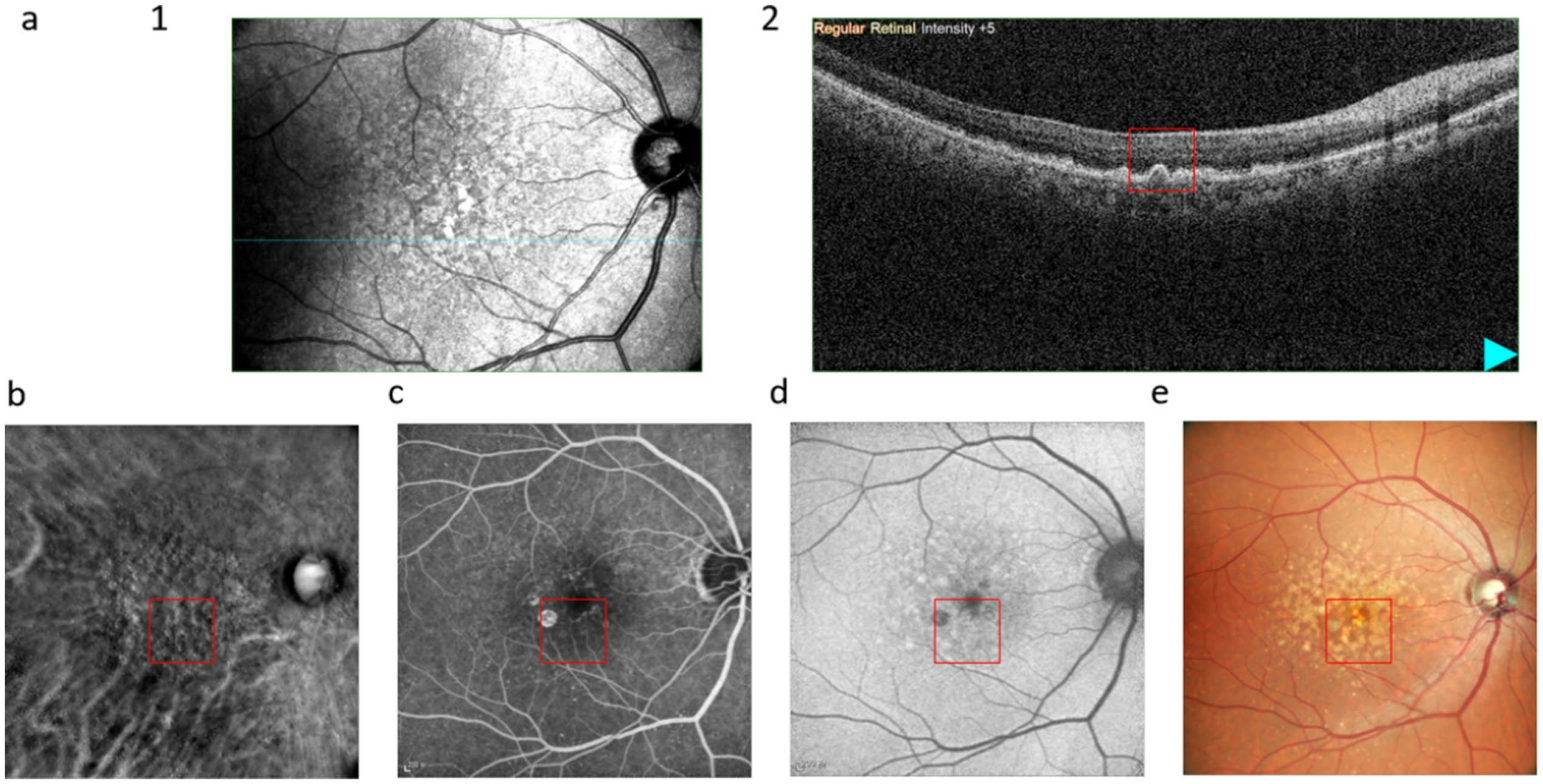
Multimodal images of soft drusen: **(a)**, (1) infrared scan, where the red line is the OCT scanning position of (2); **(b)**, RM-SLO in RMDR directions; **(c)**, FFA; **(d)**, FAF; **(e)**, CFP. The red box shows the drusen.

Figure 4 illustrates that soft drusen appear as diffuse, sandy-like soft elevations on OCT. The RM-SLO along the RMDR direction shows large depressions, whereas FFA exhibits gradually increasing fluorescence intensity over time but retains blurred borders. FAF shows low autofluorescence, and CFP manifests as irregular, large yellow masses or patches.

**Figure 5:**
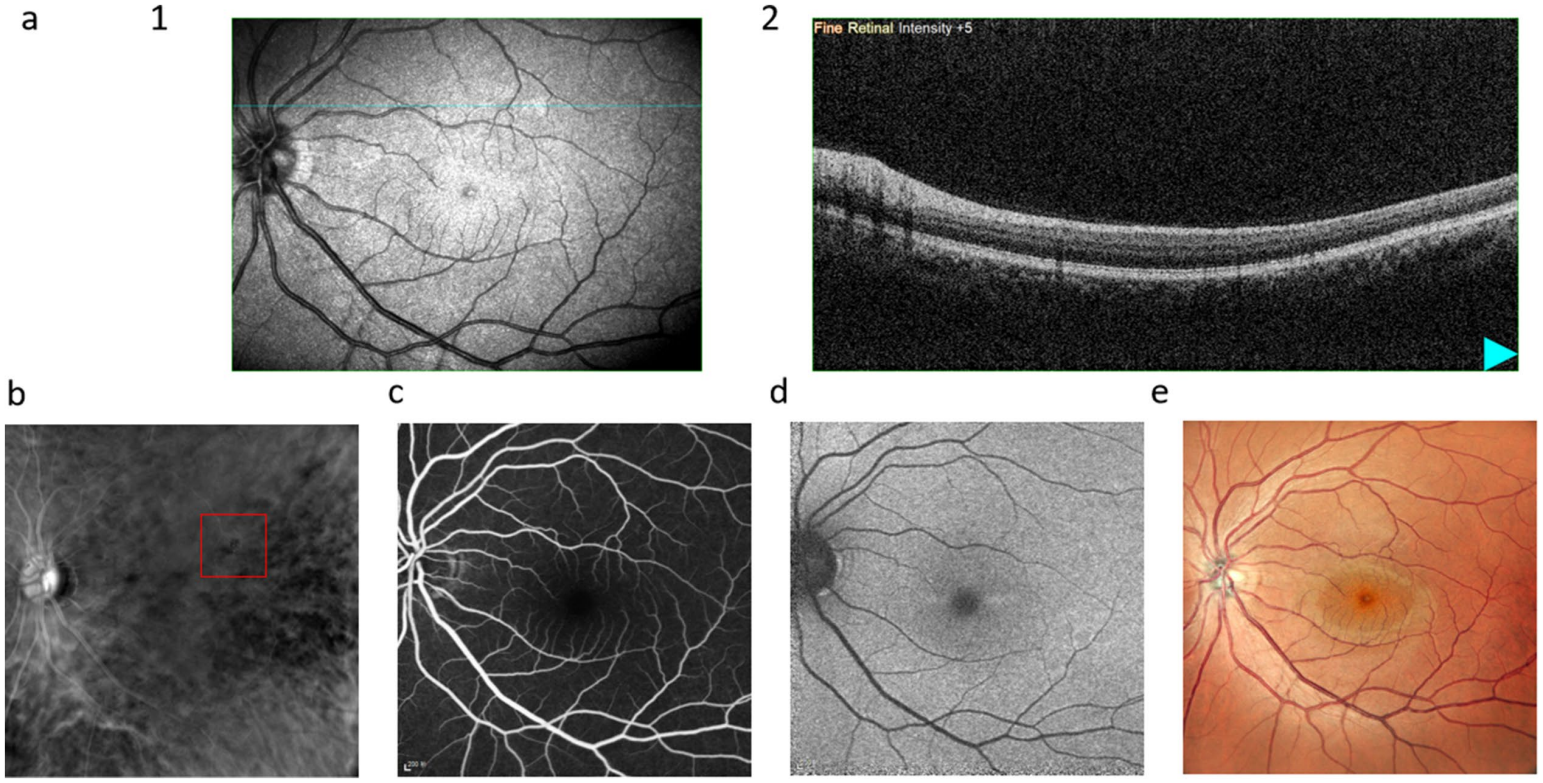
Multimodal images of drusen, which are only shown in RM-SLO: **(a)**, (1) infrared scan, where the red line is the OCT scanning position of (2); **(b)**, RM-SLO in RMDR directions; **(c)**, FFA; **(d)**, FAF; **(e)**, CFP. The red box shows the drusen.

In Figure 5, hard drusen are only visible in RM-SLO along the RMDR direction, which may be attributed to their smaller diameter, making them indistinguishable from other imaging modalities. We further summarized the main differences between OCT and RM-SLO in Table 3 to highlight their complementary roles.

**Table 3:** Comparison of OCT and RM-SLO Features.

Feature	OCT	RM-SLO
Principle	Cross-sectional imaging with interferometry	Retro-illumination with off-axis aperture
Visualization	Quantitative thickness/volume measurement	Qualitative pseudo-3D morphology
Strengths	High-resolution cross-sectional views; quantitative metrics; widely validated	High-contrast en face imaging; enhanced visualization of small drusen; non-invasive and rapid
Limitations	Limited en face information; influenced by media opacity; requires interpretation of B-scans	Primarily qualitative; potential for pseudo-artifacts; fewer standardized protocols
Clinical Use	Gold standard for structure and progression; quantitative biomarker	Promising adjunct for early detection and good screening potential

## Discussion

This study represents the first comprehensive analysis of different imaging modalities for drusen (both hard and soft drusen), revealing that RM-SLO outperforms other imaging techniques in terms of overall imaging accuracy and identification. This finding provides novel imaging insights for future drusen detection and the identification of potential early AMD risks.

Currently, the classification criteria for drusen are determined based on multimodal imaging. Hard drusen are defined as discrete yellowish-white deposits beneath the RPE, measuring < 63 μm in diameter and exhibiting a more compact structure than soft drusen. Soft drusen are defined as mound-like deposits located between the RPE and its underlying layers and the remainder of Bruch’s membrane, typically measuring ≥ 125 μm in diameter, with less distinct structural boundaries compared to hard drusen (27–29). Virgili et al. (30) demonstrated that people with drusen have a higher risk of developing AMD. Sénéclauze et al. (31) found that drusen may serve as an important biomarker for AMD progression. A meta-analysis indicates that drusen possess predictive significance and value for the progression of AMD (32). Currently, the most widely adopted approach relies on CFP for drusen detection (33). However, due to the irreversible nature of AMD and its status as one of the most severe blinding eye diseases, CFP combined with deep learning is primarily utilized for the identification and prediction of late-stage AMD. This leaves a diagnostic gap for the detection of early drusen, potential prevention, and timely intervention (34). Recent attempts to enhance drusen detection rates through additional image processing of CFP images have yielded limited results and are difficult to implement on a large scale (35). Our findings indicate that RM-SLO outperforms CFP in detecting drusen, particularly those with smaller diameters. This capability demonstrates exceptional optical sectioning capabilities. By leveraging its sensitivity to scattering characteristics at different depths, it can distinguish between the contrast from the RPE layer and Bruch’s membrane, offering the potential for identifying early-stage AMD lesions, providing a novel tool for studying early pathological changes in AMD, enabling proactive health alerts for individuals at risk, and potentially preventing future progression to AMD (36). Theoretically, it could also enable non-invasive monitoring of RPE cell changes through signal quantification, providing objective biomarkers for AMD progression and treatment efficacy—a key focus of our upcoming research (18). Although RM-SLO has shown tremendous potential in scientific research, it remains a relatively new technology. Due to the complexity of signal interpretation, RM-SLO still lacks unified image interpretation standards, large-scale clinical validation studies, and clear clinical application guidelines compared to OCT.

OCT remains the superior imaging tool for detecting lesions in the RPE layer (37). Kiruthika and Malathi et al. (38) outlined factors influencing the detection of drusen across different imaging modalities in relation to AMD progression and constructed a predictive model. The results indicated that OCT detection demonstrated a higher predictive effect (38). Overbey et al. (39) found that drusen detected by OCT are significant for the progression of AMD to macular degeneration. Our findings are consistent with previous studies. The detection rate of drusen identified via RM-SLO was not inferior to that of OCT, reflecting the excellent performance of RM-SLO in detecting RPE lesions. Additionally, not all micro-lesions are consistently detected in OCT images, which may be related to the patient’s own lens opacity. Furthermore, OCT’s cross-sectional scans are difficult to visualize in a planar manner (23). RM-SLO fundus imaging, also a non-invasive examination, offers the advantage of rapid acquisition speed. This can compensate for the limitations of cross-sectional observation in OCT examinations and is more conducive to clinically identifying the extent of drusen (23).

Shen et al. and subsequent groups have explored the use of RM-SLO for retinal diseases such as diabetic retinopathy and AMD (15, 26). While their results also suggested higher sensitivity of RM-SLO compared with CFP and OCT, methodological aspects differed. For instance, Shen et al. used RM-SLO with different aperture settings, and detection rates were calculated based on lesion counts, whereas our study relied on expert consensus diagnosis across multiple modalities. These methodological differences may partially explain variations in detection rates across studies. Another possible explanation for RM-SLO’s higher detection rates is its genuinely improved contrast and pseudo-3D rendering, which enhances the visibility of small drusen. However, one must also consider the possibility of increased false positives due to light-scattering artifacts inherent to retro-illumination. Future studies should combine RM-SLO with quantitative image analysis and histopathological validation to disentangle true sensitivity improvements from artifact-related detections.

Infrared light used in RM-SLO is not seen by the patient and, hence, permits imaging through undilated pupils and spares the discomfort that can be caused by bright flashes of other imaging modalities, such as CFP and blue autofluorescence (BAF) (36). Ranetti et al. (24) observed that the morphological presentation of drusen differed between the right and left sides based on detection rates in RM-SLO: convex on the right and concave on the left. However, such conclusions are not consistent. Cozzi et al. (25) found contrary results. Our research has yielded new findings in this regard. Detection patterns for different types of drusen in the same direction (RM-SLO) also varied. We speculate this may be related to the distinct properties of drusen: hard drusen tended to appear as raised granular structures, while soft drusen exhibited a more concave morphology (40). The specific mechanism remains unclear, but we consider it may be associated with differences in the underlying causes of drusen formation. Hard drusen tend to “deposit,” while soft drusen may “penetrate” and even coalesce, leading to neovascularization. This is why the morphological boundaries of drusen on CFP images serve as the distinguishing factor between the two types.

This study is the first to simultaneously compare the detection rates of drusen across CFP, OCT, FFA, and FAF using multimodal imaging. However, it inevitably has certain limitations: First, as a cross-sectional study, we did not conduct a follow-up to dynamically observe changes in drusen. Second, we did not classify drusen by size to investigate the sensitivity of different instruments for detecting them. Additionally, our proposed view regarding the morphology of different drusen types lacks substantial data support. Since age is a confounding factor for drusen incidence, we also consider it a potential confounding factor affecting detection rates (41). However, due to the limited research data, stratified analysis of detection rates of different detection methods in different age groups cannot be achieved at present. In the follow-up study, we will carry out screening for people of different age groups, include a sufficient sample size, and stratify the analysis of detection rates of different detection methods to improve this study. Finally, we only studied drusen as an imaging marker of early AMD and did not explore other markers. Moving forward, we will continue to collaborate with other regions to expand our dataset and explore other early AMD biomarkers through RM-SLO imaging and the mechanisms underlying these imaging differences.

## Conclusion

In summary, we have found that RM-SLO, as a novel imaging modality, demonstrates high sensitivity in detecting drusen while also compensating for the morphological and extent data lacking in OCT as a cross-sectional examination. Moving forward, we will explore the combination of multimodal imaging with AI for 3D reconstruction beyond drusen visualization, aiming to obtain more effective imaging information regarding RPE lesions.

## Data Availability

The raw data supporting the conclusions of this article will be made available by the authors, without undue reservation.
